# Development of a Whey Protein Recovery Process Using Sugar Kelp (*Saccharina latissima)* Extracts

**DOI:** 10.3390/foods13223663

**Published:** 2024-11-17

**Authors:** Alex Pierce, Denise Skonberg, Beth Calder, Rob Dumas, Qing Jin

**Affiliations:** 1 School of Food and Agriculture, University of Maine, 5735 Hitchner Hall, Orono, ME 04469, USA; alexander.pierce@maine.edu (A.P.); denise.skonberg@maine.edu (D.S.); beth.calder@maine.edu (B.C.); robert.dumas@maine.edu (R.D.); 2 Cooperative Extension, University of Maine, Orono, ME 04469, USA

**Keywords:** whey protein, alginate, carrageenan, seaweed polysaccharides, flocculation

## Abstract

Whey is the largest waste product of the cheese-making industry and the current methods of extracting the nutrients from it are costly and inefficient. This study assessed the feasibility of using crude polysaccharides to flocculate proteins from liquid whey waste. The flocculants used were a sugar kelp (*Saccharina latissima)* extract, as well as commercial seaweed polysaccharides, alginate and k-carrageenan, to recover proteins from the liquid whey waste. Physicochemical and functional parameters including protein content, protein recovery efficiency, mineral content, total phenolic content (TPC), antioxidant capacity, color, water- and oil-holding capacity, gelling capacity, foaming activity and stability, and emulsifying activity and stability were tested on the resulting flocculates. The yield of the dried flocculates by use of alginate, the sugar kelp polysaccharide extract (SKPE), and carrageenan were 1.66, 0.98, and 1.22 g/100 g of liquid whey with protein contents of 27.4%, 45.5%, and 37.5%, respectively. The protein recovery efficiency from the whey was 57.5%, 56.2%, and 57.9% using alginate, SKPE, and carrageenan, respectively. The alginate flocculate had the highest oil-holding capacity and foaming abilities while the carrageenan flocculate had the best gelling ability and the highest emulsifying activity and stability. TPC and antioxidant activity were highest in the SKPE flocculate. All three flocculates presented slightly different compositional and functional qualities, which could be used for a variety of products. This study showed that seaweed polysaccharides present a simple and effective way to extract protein from liquid whey waste while creating a functional and high-protein ingredient.

## 1. Introduction

The cheese industry generates a large amount of liquid whey, with about nine kilograms of whey produced for every one kilogram of cheese made [1]. For small- to medium-sized cheese manufacturers, most of the whey is discarded. There are methods (membrane filtration, spray drying) to recover protein from the liquid whey; however, they are typically carried out only by large cheese manufacturers because they require high initial capital investment and high energy consumption [2]. For small- to medium-sized cheese producers, it is not worth the cost of extracting whey protein. Therefore, smaller cheese manufacturers typically transport their whey to farms or to waste treatment facilities, which can be costly. For example, in 2022, Pineland Farms (the largest cheese manufacturer in Maine) paid over USD 50,000 in whey transportation fees [3]. However, only a small portion of the transported whey is upcycled as a feed supplement, with the majority incorporated into farm liquid manure management systems [3]. The flocculation of liquid whey would provide a simple, cost-effective method for small- to medium-size cheese companies to extract whey proteins and increase the utilization of this side stream.

Using this flocculation method to recover whey proteins provides a way to reduce waste while enabling resource reuse. Current global challenges include resource unsustainability, malnutrition, and high carbon emissions. The United Nations’ 2030 goals, established in 2015 to tackle these issues, remain unmet, with some showing minimal progress [4]. Although the environmental impact of the dairy industry has decreased from 2007 to 2017 [5], it is crucial to maintain and advance these improvements to prevent the loss of valuable resources. Research aimed at waste reduction is essential to foster a more sustainable food supply, supporting the development of a circular economy where all resources are maximized.

Whey byproduct is rich in essential amino acids and easily digestible proteins which represent a valuable nutrient source that would otherwise be wasted if not properly recovered. Liquid whey contains 20% of all the proteins found in milk [6], and is nutrient-dense, containing about 55% of macromolecules and minerals [7]. Liquid acid whey is composed of 5–7% total solids on average: approximately 4.18% are carbohydrates (mostly lactose); 0.84% is protein (β-lactoglobulin, α-lactalbumin, bovine serum albumin, and immunoglobulin); 0.61% is minerals; and 0.09% is lipids [8]. The total solids content of liquid whey can fluctuate depending on the season, the feed of the animals, as well as how the cheese was produced [9]; sweet whey is less acidic and mostly comes from the production of rennet-coagulated hard cheeses like cheddar and gouda while acid whey is more acidic and results from the production of acid-coagulated fresh cheeses such as cream cheese, or cottage cheese [10]. However, both sweet and acid whey are commonly mixed together into one waste stream for subsequent processing or transport, producing a consistent whey waste stream. Further, whey protein is nutritionally superior to casein and soy due to its higher protein efficiency and proportions of essential amino acids [6]. Whey protein is higher than casein in selected amino acids such as glutamine and leucine [11] and the consumption of whey protein also aids in faster stimulated protein synthesis than casein [12].

As previously mentioned, current methods of whey protein extraction are costly. Large cheese producers can recover solids, including protein, from liquid whey using membrane filtration technology, which includes nanofiltration, ultrafiltration, and crossflow microfiltration. The high costs of membrane filtration are due in part to membrane fouling. Membrane fouling often occurs because of proteins and salts, such as calcium salts, adsorbing onto the membrane and blocking the membrane pores [13]. As a result, it is hard to maintain the membrane filtration system, which makes production costs expensive. Another flaw with this technology is that it is not energy-efficient typically; membrane processing operates at an energy efficiency of 45% [2]. Spray drying is conventionally used after membrane filtration to produce whey protein powder. However, spray drying can lead to quality loss in dairy products, including whey, due to heat instability, which can cause unwanted flavors and loss in solubility [14]. Moreover, spray drying is energy-intensive due to the high heat consumption, duration of drying, and lack of energy recovery. It is estimated that up to 29% of the energy supplied to industrial spray dryers is wasted [15]. Because of these deficiencies in current whey protein extraction systems, a new process needs to be developed that is more economical and can be implemented by all cheese producers.

Liquid whey has a large environmental impact if not properly managed. Liquid whey has a high amount of organic matter as well as a high chemical and biochemical oxygen demand (above what the normal environment can support), which can contaminate water systems [16]. If it were to leach into bodies of water, it could contribute to eutrophication and algal blooms due to its nitrogen and phosphorus content, while also depleting dissolved oxygen levels and harming aquatic life [16]. Whey is also often acidic, which has the potential to harm soil microbes and reduce bioavailable nutrients, leading to reduced crop yields [17]. Providing manufacturers the ability to remove organic compounds such as protein from whey will help limit liquid whey lost to the environment due to spillage or transport. It will also reduce the amount of the organic mass that passes through wastewater treatment facilities.

While there are no previous studies performed on the use of polysaccharides for liquid whey protein flocculation, there are studies that have been performed analyzing protein–polysaccharide interactions using whey protein isolates. The focus of research on these protein–polysaccharide complexes is their structure for use in film formation [18,19] or in food matrices, commonly for emulsion stability [20,21]. There are a large variety of gels and microstructures that are formed based on the concentrations and types of the polysaccharides as well as pH, salinity, and temperature conditions [22]. There is also very limited research on the use of a crude seaweed polysaccharide extract for flocculation. The interactions from the proteins and the variety of polysaccharides are likely to create unique microstructures that will provide unique uses in foods, providing a new path for innovation. Further, there have been studies using polysaccharides to flocculate proteins from other liquid wastes. Commercial seaweed polysaccharides (e.g., alginate and carrageenan) have been previously shown to be effective in flocculating up to 78.5% of proteins in shrimp boiling water [23] and up to 70% of proteins in fish meal wastewater [24]. These studies reported the highest protein recovery with the use of carrageenan, which is predominantly found in red algae.

Sugar kelp (*Saccharina latissima)* is one of the most cultivated species of seaweed in Maine, which is the leading producer of seaweed in the United States. This seaweed contains polysaccharides that contain up to 50% of its dry weight [25]. These polysaccharides include sodium alginate, laminarins, and fucose-containing sulfated polysaccharides (FCSPs) such as fucan and fucoidan [25]. During the flocculation process, proteins are drawn towards the polysaccharide due to a variety of interactions, including electrostatic, hydrophobic, and hydrogen bonding. The proteins aggregate together with the polysaccharides and precipitate out of the solution [26]. Functional groups of seaweed polysaccharides play an important role in their interactions with proteins. For example, alginates contain numerous carboxyl groups, while carrageenan and FCSPs contain more sulfate groups [16]. These differences in functional groups can present differences in flocculation abilities.

In this experiment, we used sodium alginate, k-carrageenan, and a crude soluble polysaccharide extracted from sugar kelp as flocculants and evaluated their ability to recover protein from liquid whey. We further tested the physicochemical characteristics and functionality of the dried whey protein–seaweed polysaccharide flocculates. These tests included water- and oil-holding capacity, gelling capacity, foaming activity and stability, and emulsifying activity and stability. These tests give insight into how the flocculates may function in a food matrix and potential applications for which they could be commercially used. By integrating seaweed polysaccharide extracts in cheese production, innovative products could be created to mutually benefit both the seaweed and cheese industries.

## 2. Materials and Methods

### 2.1. Materials

Sugar kelp (*Saccharina latissima*) was provided by Gulf of Maine, Inc. (Pembroke, ME, USA) and Ocean’s Balance (Biddeford, ME, USA) and kept frozen at −30 °C until use. Liquid whey was collected on 16 November 2023 from Pineland Farms Dairy Company (Bangor, ME, USA). Alginic acid, sodium salt (sodium alginate) was purchased from MP Biomedicals (Solon, OH, USA). Carrageenan was purchased from Sigma-Aldrich (St. Louis, MO, USA). Vegetable oil was purchased from a local market. NaOH (99%) was purchased from Fisher Scientific (Waltham, MA, USA), and HCl (37%) was purchased from Fisher Scientific (Waltham, MA, USA). Mucin, alpha-amylase, pancreatin, pepsin, 2-diphenyl-1-picrylhydrazyl (99%), and Coomassie brilliant blue G-250 (99%) were also purchased from Sigma-Aldrich (St. Louis, MO, USA). Bile was purchased from Chem-Impex (Wood Dale, IL, USA). All other chemicals were of an analytical grade and were also purchased from Fisher Scientific (Waltham, MA, USA).

### 2.2. Overview of Preliminary Screening Design

Preliminary screening was conducted to determine optimal flocculation depending on the pH of the whey, polysaccharide type, concentration of the polysaccharides, and incubation time. The pH of the whey was adjusted using either 1 M NaOH or 1 M HCl to six different pH values ranging from 2 to 6, and soluble protein of the supernatant was measured to determine a pH for further testing. Alginate, SKPE, and carrageenan were the three polysaccharide types tested, and each was tested at six different concentrations ranging from 0.5 to 5 g/L. Each mixture was flocculated for 1 h and 3 h in duplicate for a total of 36 treatments and 72 samples. Treatments were tested against a base control of plain whey and a pH-adjusted control of plain whey at pH 4.5, matching the pH adjustment applied to all samples containing polysaccharides.

#### 2.2.1. Sugar Kelp Polysaccharide Extraction

Polysaccharide extraction was conducted according to Phomkaivon et al. [27]. Thawed frozen seaweed (50 g) was mixed with 360 g of room temperature (25 °C) distilled water (1.25% solids *w*/*v*) and homogenized with a commercial blender (Waring Laboratory, Torrington, CT, USA) for 6 min, followed by heating (80 °C) and mixing with a stir bar (600 rpm) for 1 h. The homogenate was then centrifuged (Beckman Coulture Avanti J-E, Brea, CA, USA) at 9700× *g* at 30 °C for 20 min. The supernatant was collected and freeze-dried for 4 h at −30 °F and 30 h at 80 °F in a freeze dryer (HarvestRight, Salt Lake City, UT, USA) to obtain SKPE powder.

#### 2.2.2. Flocculation Treatment of Whey

Flocculation treatments were modeled after the experimental design by Forghani et al. [23]. Liquid whey (20 mL) was adjusted using 1 M HCl to pH values of 2, 3, 4, 4.5, 5, and 6. This was followed by adding 10 g/L solutions of alginate, carrageenan, and SKPE to achieve final concentrations of 0.5, 1, 2, 3, 4, and 5 g/L. Each treatment was stirred for 1 min and rested for 1 or 3 h. Sediment height was measured after the flocculation process, and the supernatant was collected for a soluble protein analysis and turbidity testing.

#### 2.2.3. Soluble Protein Analysis

The protein content of the flocculated whey supernatant was measured in duplicate samples using the Bradford method [28]. The supernatant was diluted with deionized water (1:2 *v*/*v*) and 0.1 mL of the sample was mixed with 5 mL of the Bradford reagent. Absorbance was read at 595 nm using a spectrophotometer (Beckman Du 530, Brea, CA, USA) against a bovine serum albumin (BSA) standard at concentrations of 10–100 μg/mL. Protein reduction (%) in the supernatant was calculated in reference to the protein content of the plain whey.

#### 2.2.4. Turbidity

The absorbance of the flocculated whey supernatant was measured in duplicate samples using a spectrophotometer (Beckman Du 530, Brea, CA, USA) at 400 nm against distilled water to estimate the turbidity [23].

#### 2.2.5. Sediment Height

The treated whey solutions were placed into 50 mL graduated cylinders and the volume of the total solution (mL) and the volume of the sediment (mL) were measured at 0, 1, and 3 h. The sediment height percentage was calculated by dividing the sediment height by the total solution height [1]. Samples were measured in duplicate.

### 2.3. Scale-Up of Treatments

**Figure 1:**
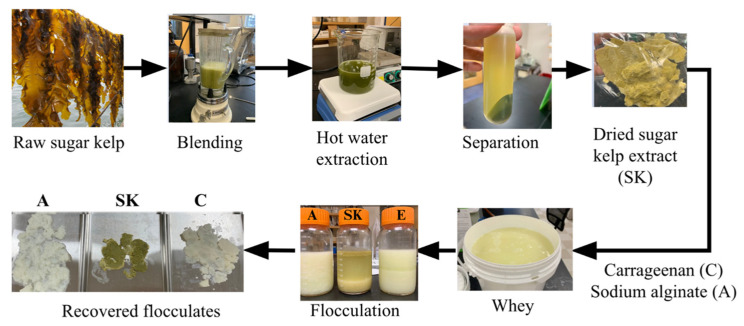
Flow chart of the process to flocculate whey protein using seaweed polysaccharides.

Based on preliminary screening of the treatments using soluble protein content, turbidity, and sediment height data, a whey pH level of 4.5, a polysaccharide concentration of 3 g/L, and an incubation time of 3 h were chosen for further investigation. As performed by Forghani et al. [23], the optimized treatment was scaled up to determine if the amount of protein flocculated out would maintain at larger amounts. Two liters of whey was adjusted to a pH of 4.5 using 1 M HCl and then divided into three, 600 mL aliquots, to which 1% solutions of carrageenan, alginate, and SKPE were added to achieve a final polysaccharide concentration of 3 g/L. After mixing, the samples were incubated for 3 h at room temperature to obtain the flocculates. The samples were then centrifuged (Beckman Coulture Avanti J-E, Brea, CA, USA) at 7000× *g* for 15 min to remove the supernatant. The flocculates were then collected, weighed, and freeze-dried for 4 h at −30 °F and 30 h at 80 °F (HarvestRight, Salt Lake City, UT, USA) for further analyses (Figure 1). This process was repeated to obtain two replicates.

### 2.4. Flocculate Yield, Protein Contents, and Protein Recovery Efficiency

Flocculate yield was determined in duplicate by measuring the weight (g) of the flocculate after freeze drying and dividing it by the starting mass of whey and reported as g of flocculate/100 g of whey.

The total nitrogen content of duplicate flocculate samples was determined by the Dumas combustion method and crude protein content (%) was estimated by multiplying the percent total nitrogen by a factor of 6.38, which is the standard for dairy products [29].

The protein content of liquid whey was determined using the method as described by Lowry et al. [30]. Briefly, a solution consisting of 2% Na_2_CO_3_ in 0.4% NaOH, 1% cupric sulfate, and 2.7% sodium potassium tartrate (100:1:1) was prepared. An aliquot (5 mL) of this solution was added to 100 µL of a sample extract and 400 µL of distilled water and incubated for 10 min. Folin’s Ciocalteu Reagent (2 N) was then diluted with distilled water (1:2) of which 0.5 mL was added to the sample solution. The sample was vortexed and then incubated for 25 min. The sample was read at 700 nm using a spectrophotometer (Beckman Du 530, Brea, CA, USA) against a standard curve of bovine serum albumin (0–0.2 mg/mL) to calculate protein content. Distilled water was used as the blank and analyses were performed in triplicate.

Protein recovery efficiency (%) was calculated by dividing the weight of protein in the flocculate (g) by the mass of protein in the initial whey sample (g).

### 2.5. Mineral Composition and Color of Flocculates

#### 2.5.1. Mineral Analysis

Analyses of Ca, K, Mg, P, Na, and Fe contents of duplicate flocculate samples were conducted using the methods of Shearer et al. [31]. Flocculates (1 g) were placed in 20 mL scintillation vials and heated at 100 °C for 6 h. The samples were then heated at 550 °C for 6 h. The resulting ash was dissolved in 1 mL of nitric acid and 1 mL of hydrochloric acid, and then brought up to 100 mL with distilled water. The samples were then analyzed using inductively coupled plasma optical emission spectroscopy (ICP-OES) (Thermo Elemental IRIS Intrepid ICP-OES, Thermo Fisher Scientific, Waltham, MA, USA) by the radial mode against standard curves of each mineral tested and reported as mg of mineral/100 g of flocculate.

#### 2.5.2. Color

L*, a*, and b* values of dried flocculates were measured using a colorimeter calibrated with white and black tiles using the D65 setting (LabScan XE, Hunter Labs, Reston, VA, USA). The flocculates (2 g) were placed into 1.75 mm diameter plastic cups. The samples were measured by rotating the sample cup 120° twice for a total of three readings of each color value. These values were then averaged to determine the mean L*, a*, and b* values. The Browning index (BI) was calculated from L*, a*, and b* values using the formulas as described by Kasim and Kasim [32].

### 2.6. Functionality Testing of Seaweed Polysaccharide Flocculates

#### 2.6.1. Water- and Oil-Holding Capacities (WHC and OHC)

The methods used were followed from a previously published paper [33]. The flocculate (0.1 g) was mixed with 1.5 mL of vegetable oil or deionized water and vortexed for 1 min. The sample was incubated at room temperature for 30 min and then centrifuged at 5000× *g* for 30 min. The supernatant was discarded, and the remaining sample was reweighed. Results were reported as g of oil or water bound per g of sample. This was measured in duplicate.

#### 2.6.2. Gelling Capacity

Gelling capacity of duplicate samples was evaluated as described by Aydemir and Yemenicioğl [34]. Solutions of 3–15% flocculate in distilled water (*w*/*v*) were prepared and 2 mL of each were deposited into test tubes. The test tubes were heated at 90 °C for 1 h and then incubated for 2 h at 2 °C. The test tubes were inverted and the least gelling capacity (LGC) was determined as the lowest concentration (% protein) at which a hard gel formed without slipping when inverted.

#### 2.6.3. Emulsifying Activity and Stability

Emulsifying activity and stability were determined using previously described methods [35]. A 0.2% solution (*w*/*v*) of the flocculate in deionized water (24 mL) was homogenized for 30 sec with 8 mL of vegetable oil in a commercial blender (Waring Laboratory, Torrington, CT, USA). At 0, 5, 10, 15, 20, 30, and 60 min after homogenization, a 50 μL aliquot from the bottom of the emulsion was vortexed with 4.95 mL of 0.1% (*w*/*v*) sodium dodecyl sulfate solution and absorbance was read on a spectrometer (Beckman Du 530, Brea, CA, USA) set at 500 nm against a blank of deionized water. This was measured in duplicate samples.

#### 2.6.4. Foaming Activity and Stability

Foaming activity and stability were determined as described by Aydemir and Yemenicioğl [34]. Duplicate flocculate solutions (10%, *w*/*v*) were prepared in distilled water and homogenized in a commercial blender (Waring Laboratory, Torrington, CT, USA) for 1 min. Homogenized samples were poured into a 100 mL graduated cylinder and volumes (mL) of the foam and the solution were recorded. Foaming activity (%) was calculated by dividing the volume of the foam (mL) by the total solution volume (mL). Foam stability was determined by comparing the foaming activity (%) at 30 and 180 min.

### 2.7. Antioxidant Assays

#### 2.7.1. Sample Preparation

Freeze-dried flocculate samples (2 g) were extracted in duplicate with 20 mL of 60% methanol for 24 h at room temperature on an orbital shaker (Fisher Scientific, Waltham, MA, USA) at 200 rpm. The samples were then centrifuged at 2000× *g* for 10 min. The supernatant was collected and the pellet washed twice by adding 10 mL of 60% methanol and vortexing for 2 min and centrifuging at 2000× *g* for 10 min. The supernatants were commingled, brought to 50 mL with distilled water, and vortexed for 30 s.

#### 2.7.2. Total Phenolic Content (TPC)

Total phenolic content of extracts was determined using adapted methods from Islam et al. [36]. The Folin–Ciocalteu reagent (2 N) was diluted with distilled water (1:10 *v*/*v*). The diluted reagent (1.5 mL) was added to a 200 μL aliquot of the sample extract and vortexed. The samples were incubated for 5 min at room temperature and 1.5 mL of 6% (*w*/*v*) sodium bicarbonate solution was added to the sample and vortexed. Samples were then incubated for 1 h in the dark. The samples were blanked against 40% methanol, and absorbance was measured at 750 nm (Beckman Du 530, Brea, CA, USA). A standard curve (0–250 μg/mL) of gallic acid was used to estimate TPC, which was expressed as mg gallic acid/g of freeze-dried sample. Samples were measured in duplicate.

#### 2.7.3. DPPH (2-Diphenyl-1-Picrylhydrazyl) Assay

DPPH radical scavenging activity was determined using Blois’ method [37] with modifications. A 0.2 mM DPPH solution was prepared in 99.8% ethanol. Volumes of the sample extract (0.5–2 mL) were brought up to 2 mL with 40% methanol and 2 mL of the DPPH solution was added. The sample was vortexed and incubated for 30 min in the dark. Sample blanks were prepared similarly with 2 mL of 99.8% ethanol instead of the DPPH solution. The control was 40% methanol and either 2 mL DPPH or ethanol was added to 2 mL 40% methanol. Absorbance was measured against 100% ethanol at 517 nm and plotted against sample concentrations to determine EC_50_, the concentration of the sample needed to quench 50% of the DPPH free radical. Ascorbic acid was used as a positive control. The assay was performed in duplicate, and the average was expressed as EC_50_ (mg/mL).

### 2.8. Protein Digestibility

Protein digestibility was achieved according to the methods by Świeca et al. [38]. Three solutions were made to mimic phases of digestion including salivary, gastric, and intestinal breakdown. The saliva solution was prepared by mixing 100 mg mucin (derived from porcine stomach), 2.38 g of sodium phosphate, 0.19 g of potassium phosphate, and 8 g of sodium chloride in 1 L of distilled water. The pH of the solution was then adjusted to 6.75 and α-amylase (from *Aspergillus oryzae)* was added to produce an enzyme activity of 200 U/mL. The next solution prepared was the gastric juice, which contained pepsin (≥250 U/mg; from porcine gastric mucosa), at an enzyme activity of 300 U/mL in 0.03 M sodium chloride. This solution was adjusted to a pH of 1.2 using 2 M HCl. The last solution of the series was the imitation intestinal juice, which was produced by dissolving 0.125 g of pancreatin (8 × USP; from porcine pancreas) and 1.5 g of bile (from ox gall) in 0.1 M sodium bicarbonate.

The freeze-dried flocculates (1 g) were each put into stomacher bags with 15 mL of the saliva solution and massaged for 1 min to simulate mastication. The samples were then shaken for 10 min at 37 °C in a reciprocal water bath (Sheldon Manufacturing, Cornelius, OR, USA). The samples were adjusted to a pH of 1.2 using 2 M HCl and 15 mL of the gastric juice was added. The samples were shaken at 37 °C for 60 min in the water bath. The pH of the samples was adjusted to a pH of 6 using 0.1 M sodium bicarbonate before the addition of 15 mL of the intestinal juice solution. The solution was adjusted to a pH of 7 with 1 M NaOH and then 5 mL of 0.12 M sodium chloride and 5 mL of 0.12 M potassium chloride were added. The samples were incubated in the dark at 37 °C for 120 min. The final solution was centrifuged at 5000× *g* for 10 min.

The protein content of the supernatant was analyzed by the Bradford method [28] (see Section 2.2.3), and protein digestibility % was calculated by dividing the soluble protein content and dividing by the starting protein content of the flocculates and subtracting that value from 100%. This was performed in duplicate.

### 2.9. Fourier Transform Infrared Spectroscopy

FTIR was used to analyze the functional groups of the raw and flocculated products based on methods adapted from Kim et al. [39]. The freeze-dried flocculates and freeze-dried whey powder were placed on an attenuated total reflectance crystal and pressure was applied to form films. The films were analyzed using an FTIR spectrophotometer (Spectrum 2 FTIR spectrophotometer, Perkin Elmer, Waltham, MA, USA) within a spectral range of 400–4000 cm^−1^ with 32 scans per sample with a spectral resolution of 4 cm^−1^. Spectra were analyzed using PerkinElmer Spectrum^TM^ 10 spectrum software and background noise was eliminated.

### 2.10. Statistical Analysis

Data were analyzed using SPSS v29. A one-way analysis of variance (ANOVA) was selected for comparing differences among treatments with Tukey’s Honest Significant Difference (HSD) test used for post hoc analyses. A significance level of *p* < 0.05 was the limit for all the statistical analyses. Color, oil- and water-holding capacity, and emulsification activity and stability were evaluated in triplicate and the rest of the tests were performed in duplicate.

## 3. Results and Discussion

### 3.1. Flocculation Efficiency

#### 3.1.1. Protein Recovery

The flocculation efficiency of treatments was based upon (1) turbidity of the supernatant from the flocculation process, whereby a lower turbidity indicates less refracted light and fewer solids in the supernatant, which indicates higher rates of flocculation; (2) sediment height, which estimates the amount of solids that precipitated out of the solution due to reduced solubility or flocculation; and (3) protein content of the supernatant, where the soluble protein reduced by treatment was determined.

**Figure 2:**
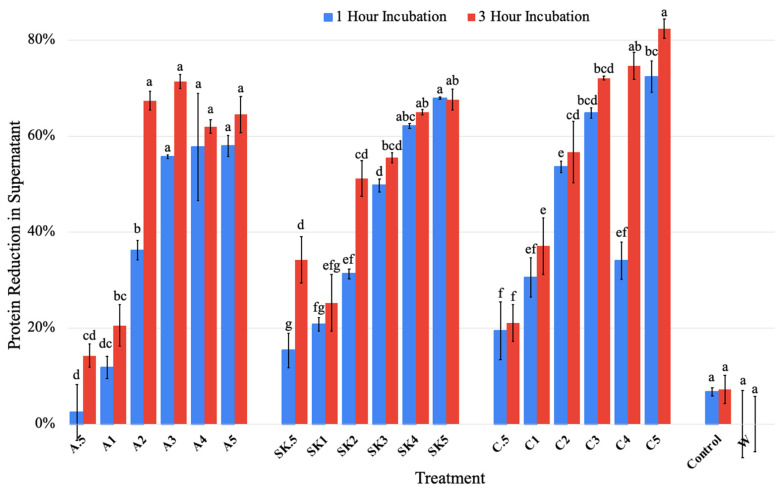
Mean percent soluble protein reduction +/− standard deviation in supernatants of treated samples after 1 and 3 h. A = Alginate; SK = SKPE; C = Carrageenan; Control = pH-adjusted whey; W = Plain whey; 0.5–5 = Concentration (g/L) of polysaccharides. Data were statistically analyzed by ANOVA (*p* < 0.05) within polysaccharide treatments. Differences in lowercase letters within treatment sets denote significant differences among treatments.

The first independent variable tested was the whey pH level, which was adjusted from 2 to 6 from its original pH of 5.2. An adjustment of the whey pH level down to 4.5 led to the lowest soluble protein content of the supernatant, so that value was used as the basis for the subsequent trials with the addition of polysaccharides. Adjustment of the pH value closer to the isoelectric point of a protein causes the net charge of the protein to approach 0, making it less polar and reducing its interactions with water, causing the precipitation of protein out of the solution [40]. The isoelectric point of the proteins in liquid whey mostly falls between pH values of 4.5 and 5.5 [41], with one study finding adjustment to pH of whey to 4.6 leading to minimal protein solubility [42]. This is mostly due to the isoelectric point of β-lactoglobulin, which makes up 50% of the proteins in whey [42]. If the whey remained at its starting pH (5.2), the proteins would not be positively charged as the pH would remain above their isoelectric point. This would reduce the electrostatic attractions between the whey proteins and polysaccharides [26]. From pH adjustment of the whey from 5.2 to 4.5 alone, protein in the supernatant was reduced by 7% (Figure 2). While pH adjustment does not provide significant (*p* < 0.05) protein sedimentation, it is still used for further testing as it is a large proponent of electrostatically induced protein aggregation [19].

All polysaccharide treatments other than 0.5 g/L alginate had a significantly (*p* < 0.05) greater reduction in soluble protein content in the supernatant than from pH adjustment alone. The greatest reduction in protein in the supernatant was 82%, which was for the carrageenan treatment at 5 g/L for 3 h. The crude sugar kelp extract (5 g/L) resulted in a 68% reduction after 3 h, similar to the results for the alginate (65%).

Protein reduction in the supernatant was directly related to protein recovery. As for flocculation time, protein reduction in the supernatant after incubation for 3 h was significantly (*p* < 0.05) higher than after 1 h for carrageenan at 4 and 5 g/L as well as alginate and SKPE at 2 g/L (Figure 2). Due to these results, a flocculation time of 3 h was chosen to continue with scale-up. This is consistent with another study that found that a longer flocculation treatment time was shown to be more effective in extracting proteins from shrimp boiling water [23].

As the concentration of the polysaccharides increased, more protein was reduced in the supernatant except for a few outliers. However, there was no significant (*p* > 0.05) effect above 2, 3, and 4 g/L, for the alginate, SKPE, and carrageenan treatments, respectively, of polysaccharide concentration on protein precipitation after 3 h of flocculation.

#### 3.1.2. Sediment Height Analysis

**Figure 3:**
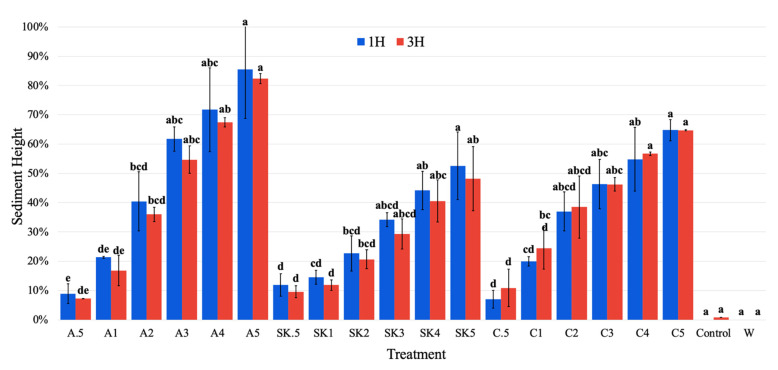
Mean sediment height of treated samples after 1 and 3 h +/− standard deviation. A = Alginate; SK = SKPE; C = Carrageenan; Control = pH-adjusted whey, W = Plain whey; 0.5–5 = Concentration of polysaccharides in g/L. Data were statistically analyzed by ANOVA (*p* < 0.05) within polysaccharide treatments. Differences in lowercase letters within treatment sets denote significant differences among treatments.

Sediment height is an estimate of the percentage of total solution height that was taken up by sediment. A higher value suggests that more solids flocculated/precipitated from the solution, but that assumption is not always valid since the sediment can compact, which affects the sediment height. At polysaccharide concentrations of ≥3 g/L, alginate treatments had consistently higher sediment heights than the other polysaccharide treatments due to their tendency to float (Figure 3). This buoyancy of alginate gels has been used in the development of floating microbeads by cross-linking with calcium [43]. Carrageenan treatments had higher sediment heights than the SKPE treatments; however, there was no significant (*p* > 0.05) effect of incubation time (1 h vs. 3 h) on sediment heights among samples.

As polysaccharide concentrations increased, sediment height also increased. However, there was no significant (*p* > 0.05) increase in sediment height for polysaccharide concentrations above 3, 2, and 2 g/L for alginate, SKPE, and carrageenan, respectively, after 3 h. A greater concentration of polysaccharides leads to the chance of more interaction between molecules, leading to more sedimentation.

#### 3.1.3. Turbidity Analysis

**Figure 4:**
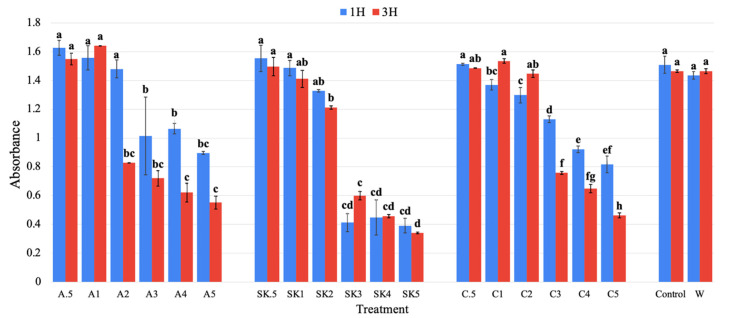
Turbidity (absorbance +/− standard deviation) at 400 nm of treated samples after 1 and 3 h. A = Alginate; SK = SKPE; C = Carrageenan; Control = pH-adjusted whey; W = Plain whey; 0.5–5 = Concentration of polysaccharides in g/L. Data were statistically analyzed by ANOVA (*p* < 0.05) within polysaccharide treatments. Differences in lowercase letters within treatment sets denote significant differences among treatments. The results of these three preliminary tests were used to determine the subsequent scale-up conditions of 3 g/L of polysaccharides for 3 h after a whey pH adjustment of 4.5. The incubation time was selected due to the significant (*p* < 0.05) differences in turbidity and protein reduction in some samples from 1 h to 3 h. Polysaccharide concentration was chosen based on the lowest concentrations that were not significantly (*p* > 0.05) different from 5 g/L. While these screening tests helped evaluate the extent of flocculation and protein reduction in the supernatant, they did not provide any insight into the protein versus polysaccharide composition of the flocculate.

The turbidity of the supernatants provides an indirect measure of the amount of the concentration of suspended proteins resulting from flocculation; as protein is flocculated out, turbidity correspondingly decreases (Figure 4). The SKPE treatment was observed to have the lowest turbidity at concentrations of 3–5 g/L compared to the other polysaccharide treatments. There was a general trend for turbidity to decrease with an increase in polysaccharide concentration, as expected. However, the large and significant (*p* < 0.05) jump in turbidity between treatments at concentrations of 2 g/L and 3 g/L was particularly interesting and suggests better protein–polysaccharide interactions at certain levels. A similar trend was reported in a study involving carrageenan for gelling whey protein isolate aggregates, where turbidity sharply increased from 0 to 0.5 g/L and then increased gradually as the concentration was increased up to 5 g/L, aligning with the trends observed in this experiment [44]. There were significant (*p* < 0.05) differences in turbidity between 1 and 3 h for carrageenan at a concentration of 3–5 g/L and for alginate at concentrations of 2–3 g/L. However, increased incubation time did not result in reduced turbidity for the SKPE treatments, showing that most of the protein flocculation occurred within the first hour. The turbidity of the pure alginate, sugar kelp extract, and carrageenan solution was also tested, resulting in 0.179, 0.243, and 0.201, respectively.

### 3.2. Flocculate Properties

#### 3.2.1. Yield, Protein Content, and Protein Recovery Efficiency

The average yields of the dried flocculates were 1.66, 0.98, and 1.22 g/100 g of liquid whey, and the protein concentrations of these flocculates were 27.4%, 45.4%, and 37.5%, for the alginate, SKPE, and carrageenan flocculates, respectively (Table 1). The higher flocculate mass and lower protein concentration of the alginate flocculate were likely due to increased interactions between alginate and lactose as compared to the other polysaccharides, causing more lactose to flocculate with the protein. The major compound in whey that is abundant enough to cause such substantial differences in flocculate weight is lactose [9], and alginates have previously been shown to form strong gels that readily trap lactose [45]. The binding capacities of the carboxyl groups in alginates and the sulfate groups present in carrageenan and SKPE dictate their effectiveness in binding to other molecules such as proteins or sugars [46].

The whey protein recovery efficiencies were 53.8%, 52.6%, and 54.2% for the alginate, SKPE, and carrageenan flocculates, respectively. There was no significant (*p* > 0.05) difference among these values. Polysaccharide–protein, electrostatic interactions are very dependent on pH; a negatively charged polysaccharide can form ionic bonds with positively charged proteins, meaning that pH values above a polysaccharide’s isoelectric point will be more effective in facilitating the interactions with, and flocculating out of, proteins [47]. Due to the carboxyl groups within the structure of alginate, which have an isoelectric point around 3.5, and the sulfate groups in the structures of carrageenan and SKPE, which have an isoelectric point of 2, the polysaccharides will have negative charges in the presence of the acidified whey (pH 4.5), causing their deprotonation [44,45]. At a pH of 4.5, which is lower than the isoelectric point of whey proteins, these proteins will be slightly positively charged. The negatively charged polysaccharides and positively charged proteins will then be drawn to each other, causing the aggregation of the proteins. Carrageenan and SKPE would be expected to have increased ionic binding with the proteins in comparison to alginate, since a pH of 4.5 is farther from their isoelectric points.

#### 3.2.2. Mineral Content Analysis

Mineral content was assessed to determine if any minerals from the sugar kelp were retained in the SKPE flocculate and to establish whether the flocculates were mineral-rich enough to promote their use from a nutritional perspective. The SKPE flocculate was hypothesized to contain higher amounts of the minerals prevalent in seaweed, in comparison to the treatments using purified seaweed polysaccharides. The SKPE flocculate was significantly (*p* < 0.05) higher in iron than the other flocculates (Table 1). Iron is a predominant trace mineral found in sugar kelp [48], suggesting that the iron was transferred from the seaweed. However, kelp is also well known to be high in potassium, calcium, magnesium, sodium, and phosphorus [49], none of which were demonstrated to be significantly higher in the SKPE flocculate. The SKPE flocculate seemed to not retain any of these minerals from the kelp, in fact having a significantly (*p* < 0.05) lower amount of potassium compared to the other two flocculates. The minerals from the kelp were likely lost during the extraction process during the removal of the seaweed pellet after centrifugation. The potassium content of the carrageenan and alginate flocculates came from the whey itself since they were purified. The higher amount of potassium in the purified polysaccharide treatments could be due to a higher affinity of these polysaccharides to potassium. The alginate flocculate was significantly (*p* < 0.05) higher in sodium than the other flocculates, as expected, since sodium alginate was used in the flocculation process. Another interesting observation is that the alginate, SKPE, and carrageenan flocculates had approximate calcium-to-magnesium ratios of 10:1, 8:1, and 4:1, respectively, which were higher than the typical mineral ratio found in seaweed (a 2:1 calcium-to-magnesium ratio [50]). This could be attributed to the high calcium content in whey, as whey typically shows calcium-to-magnesium ratios of approximately 10:1 in acid whey and 5:1 in sweet whey [51].

#### 3.2.3. Color Analysis

Color was measured to determine the impact of the flocculation process on appearance and specifically, whether any kelp pigments would leach into the flocculate. Colors, such as those imparted by sugar kelp, could limit the applications of these flocculates in light-colored formulated products. The SKPE flocculate had a deep greenish-brown color imparted by the seaweed extract while the alginate and carrageenan flocculates were gray-white in color. The average L* value of the SKPE flocculate was significantly (*p* < 0.05) lower than L* values of the other two treatments, as it had a visibly darker color (Table 2). The a* value was positive for the SKPE flocculate, signifying a more reddish color compared to the other two treatments. The b* value was also significantly (*p* < 0.05) higher for the SKPE treatment, indicating more yellow undertones. The SKPE flocculate was found to have a significantly (*p* < 0.05) higher browning index. These color differences show that the pigments from the crude sugar kelp extract were partially recovered in the flocculate. The brown color of sugar kelp is attributed to the carotenoid fucoxanthin, which degrades with heat [52], allowing green chlorophyll compounds to be more visible in the flocculate, conferring a green color [53]. The alginate and carrageenan used were from commercial sources and were observed to be purified white powders that added no color to the flocculate. Liquid whey is slightly yellow-green due to riboflavin, which can degrade by heating [54], and which also causes the yellowing of whey powder [1], leading to the carrageenan and alginate flocculates being a pale beige (Table 2). The SKPE flocculate would likely perform best where its color would blend in or be masked, such as in a smoothie or savory dish; however, its color limits potential food applications. The more neutral color of the carrageenan and alginate flocculates would likely not limit their applications in a wide variety of formulated foods.

### 3.3. Functionality

#### 3.3.1. Water-Holding Capacity Analysis

WHC is a measure of the amount of water that can be bound to the flocculate. WHC can be important for dough hydration in baked goods as well as impacting the viscosity and mouthfeel of sauces. The alginate flocculate had significantly (*p* < 0.05) higher WHC than the SKPE and carrageenan flocculates (Table 3). This was consistent with a study that showed that WHC of blue whiting mince was higher with the addition of alginate than it was with carrageenan [55]. The WHC of the SKPE and carrageenan flocculates were not significantly (*p* > 0.05) different. The presumed high content of lactose in the flocculate could be the reason for the high WHC as lactose is very hydrophilic [56]. Further investigations to quantify the lactose and polysaccharide content of the flocculates would increase understanding of these functional properties.

#### 3.3.2. Oil-Holding Capacity Analysis

OHC of protein-rich ingredients is important for maintaining emulsions and thus improving the texture of the product it will go into. It is also important for holding onto flavor compounds. Both the alginate and carrageenan flocculates had significantly (*p* < 0.05) higher OHC than the SKPE flocculate (Table 3). Seaweed polysaccharides are hydrophilic and do not readily interact with oil [57]; thus, most of the oil-holding capacity of the flocculates was provided by the lactose and proteins from the whey. The oil-holding capacity of the whey protein isolate was reported as ~4 g/g [58], like the results of the SKPE flocculate, which had the highest whey protein content. The differences in OHC of the flocculates could have been due to the hydrophobicity of the flocculate components as well as the flocculate composition. Hydrophobic compounds are more likely to bind with oil. A study found that chickpea flour had a higher OHC in comparison to pea and lentil flour, which was attributed to a greater proportion of non-polar side chains and hydrophobic amino acids [59]. The differences in hydrophobicity of the flocculates due to their unique protein and carbohydrate profiles, as well as the accessibility of the hydrophobic structures to the oil, also could have affected the OHC of the flocculates.

#### 3.3.3. Gelling Capacity Analysis

All flocculates had similar gelling capacities with the lowest LGC (7%) observed for the carrageenan treatment and the highest for the SKPE flocculate (9%). A lower LGC indicates that lower concentrations of the carrageenan flocculate were needed to form a firm gel in comparison to the alginate or SKPE flocculates. Carrageenan forms strong gels in the presence of minerals such as potassium, which are found in dairy and seaweed as well as by being heated [60]. Alginate forms strong gels in the presence of divalent cations such as calcium [61], which are found in whey and in the alginate flocculate. The lower gelling capacity of the protein-rich SKPE flocculate could be attributed to a lower content of polysaccharides due to its purity, which can reduce gel strength.

#### 3.3.4. Emulsifying Activity Analysis

**Figure 5:**
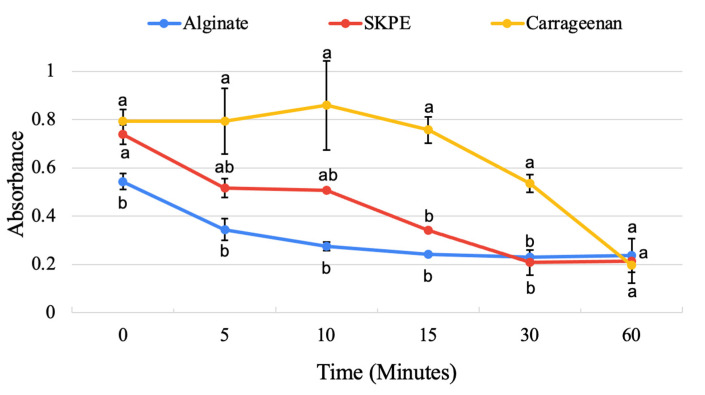
Average emulsion stability ± standard deviation over time. Data were statistically analyzed by ANOVA (*p* < 0.05). Differences in lowercase letters denote significant differences between treatments within each time.

Emulsifying activity and stability are important functions in a food system as they help keep products from separating, improving quality and texture. The initial emulsifying activity of the SKPE and carrageenan flocculates was significantly (*p* < 0.05) higher (Table 3, Figure 5) than the emulsifying activity of the alginate flocculate, as indicated by their higher absorbance values characteristic of numerous small oil droplets in a solution [62]. Smaller droplets also have better emulsion stability. Carrageenan has been reported to increase emulsion stability by increasing the viscosity of the mobile phase, which has been shown to prevent oil droplets from coalescing in milk [63] and increase the stability of emulsion gels containing sunflower oil [64]. Similarly, fucoidan has also been demonstrated to modulate lipid droplet aggregation in whey protein emulsions according to this principle [65]. As for stability, the carrageenan emulsion started to decrease in absorbance from 10 min onward; however, it maintained higher absorbance values than the other flocculate solutions until 60 min (Figure 5). Emulsion stability by whey protein isolates has also been shown to be increased by the addition of carrageenan [66].

The absorbance of the alginate and SKPE flocculate emulsions immediately decreased from 0 to 5 min and while the alginate solution continued to decrease, the SKPE solution had stable absorbance values from 5 to 10 min before decreasing further.

#### 3.3.5. Foaming Activity Analysis

**Figure 6:**
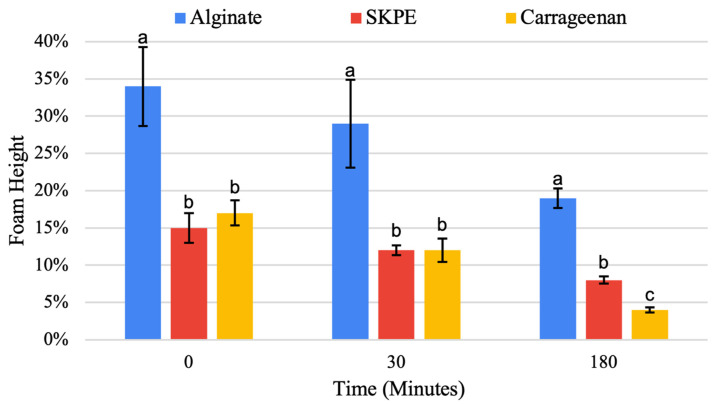
Average foam height over time +/− standard deviation. Data were statistically analyzed by ANOVA (*p* < 0.05). Differences in lowercase letters within parameters denote significant differences.

Foaming capabilities of ingredients are also an important function since foaming is desirable in aerated products such as ice creams, baked goods, and egg substitutes. The initial foaming activity of the alginate flocculate was significantly (*p* < 0.05) higher than that of the other flocculates (Table 3; Figure 6). Alginate has shown good foaming abilities previously and alginate solutions have been used to trap air as well as form hydrogel foams with CO_2_ [67]. Whey protein and alginate foam matrices with the addition of a licorice root extract were also reported to successfully maintain volume in ice cream with sugar and fat replacement levels of 25% [68]. The enhanced foam stability was due to the increase in viscosity provided by alginate, which has been found to produce smaller, more stable foam bubbles [69]. Alginate–whey protein mixtures have the potential to be used in food products such as ice cream due to their considerable foaming abilities.

As for stability, the alginate flocculate showed a significantly (*p* < 0.05) greater foam stability over all time points compared to SKPE and carrageenan flocculates and resulted in the best foam retention after 180 min (Figure 6). Foam stabilities for the SKPE and carrageenan flocculates were not significantly (*p* > 0.05) different until 3 h, at which time the carrageenan-stabilized foam had significantly (*p* < 0.05) less volume. Reduction in foam stability has previously been demonstrated where the addition of carrageenan weakened the stability of egg white foams [70]. The polysaccharides were also tested on their own but showed breaking of the emulsions at 10 min and lower absorbance values than the flocculates. This shows that the whey proteins have emulsification properties in the flocculates.

#### 3.3.6. TPC and Antioxidant Activity Analysis

The TPC of the flocculates was measured to quantify whether phenolic compounds were retained in the flocculate from the seaweed. As anticipated, the SKPE flocculate was significantly higher (0.30 mg GAE/g) in TPC than the other two flocculates (Table 3). Sappati et al. reported a TPC of 0.565 mg GAE/g in freeze-dried sugar kelp [71], which was higher than the values of the SKPE flocculate, likely due to the protein and lactose content of the flocculate provided by the whey, which is not as dense in phenolic compounds as seaweed.

Similarly, the SKPE flocculate also had the highest antioxidant activity as indicated by its significantly (*p* < 0.05) lower EC_50_ value (40.8 mg/mL) as compared to the other treatments. A lower EC_50_ represents that less of the sample is needed to quench the DPPH radicals in the assay. The higher antioxidant activity of the SKPE could possibly be a result of phenolic and other antioxidant compounds, such as carotenoid pigments, that were carried over from the sugar kelp into the flocculate. A previous study reported the EC_50_ of freeze-dried sugar kelp seaweed as 11.05 mg/mL [71], much lower than the EC_50_ value of the SKPE flocculate, again likely due to the diluting effect of non-antioxidant components precipitated from the whey. Whey proteins, specifically β-lactoglobulin, do have some antioxidant activity as shown by their ability to reduce oxidation in cooked pork patties [72]. The significant (*p* < 0.05) difference between the carrageenan and alginate flocculates may have been related to the higher concentration of proteins in the carrageenan flocculate, including greater amounts of β-lactoglobulin, contributing to its higher DPPH scavenging activity. The antioxidant abilities of just the polysaccharides were also tested and showed low antioxidant activity, meaning that the whey proteins provided antioxidant effects in the flocculates.

#### 3.3.7. Protein Digestibility Analysis

Protein digestibility was tested to determine whether the polysaccharides affected whey protein digestion following the flocculation process. Significant (*p* < 0.05) differences were observed between the pure whey powder and all the flocculates (Table 4). Seaweed polysaccharide-flocculated proteins showed significantly (*p* < 0.05) lower digestibility compared with the whey protein, which was in accordance with previous studies that anionic polysaccharides, like the ones used in these experiments, were also found to reduce proteolysis [73]. In addition, research showed that polysaccharides increase stomach viscosity, which has been shown to reduce protein digestibility by decreasing enzymatic activity [74]. The SKPE flocculate had the highest concentration of polysaccharides, followed by the carrageenan and alginate flocculates, which could explain the corresponding decrease in digestibility due to higher viscosity with increased polysaccharide concentrations. The lower digestibility of the SKPE flocculate could also be explained by the presence of proteins or fibers derived from the seaweed itself, which are known for their low protein digestibility [75]. Although protein digestibility was reduced, this effect may contribute to increased satiety as the proteins are digested more slowly [76].

#### 3.3.8. FTIR Analysis

**Figure 7:**
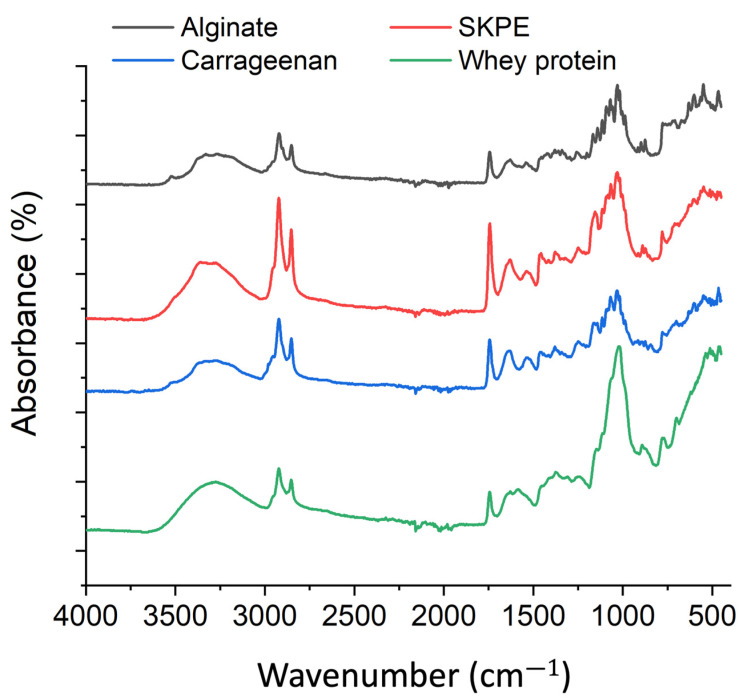
FTIR of the raw whey protein and seaweed polysaccharide-flocculated proteins.

The functional groups of the raw and flocculated proteins are shown in Figure 7. The raw whey protein showed high intensity peaks around 1020–1220 cm^−1^, attributed to alkyl amine, which primarily originated from protein [77]. After flocculating with polysaccharides, the peak intensity within 1020–1220 cm^−1^ decreased. The peak at 1749 cm^−1^ is caused by the stretching vibrations of C=O ester bonds [78]. The peak at 1535 and 1629 cm^−1^ corresponded to amide I and II, respectively. Amide I is associated with C=O and C-N stretching, while amide II is linked to N-H bending and C-H stretching [79]. Since nitrogen content can be a reliable indicator of protein levels, the data showing that SKPE exhibits the highest intensity across amide I and II suggest a higher protein content in its flocculate (Figure 7). This aligns with the protein analysis results (Table 1). Variations in the seaweed polysaccharide composition likely explain the differences observed at 700–900 cm^−1^ [80].

## 4. Conclusions

This study investigated the effectiveness of using seaweed polysaccharides to extract proteins from whey, a common byproduct from cheese producers. Protein recoveries of 57.5%, 56.2%, and 57.9% for alginate, crude sugar kelp extract, and carrageenan flocculates showed that this process presents a promising opportunity for small- to medium-sized cheese manufacturers to recover lost whey protein, without having to invest in expensive whey protein recovery equipment. Higher concentrations of the polysaccharides for flocculation may produce even better protein extractions on a large-scale basis. The retained sugar kelp phenolics, antioxidant activity, and color in the SKPE flocculate present an interesting opportunity to be used as a protein-rich ingredient that could be suitable in savory applications such as a flavored spread or entrée (e.g., filled pasta). Meanwhile, the alginate flocculate, due to its foaming abilities and water- and oil-holding capacities, may be useful in dairy products, including high-protein dips or beverages, without imparting residual seaweed color and flavor. The carrageenan flocculate also has potential as an ingredient for select products as it showed beneficial emulsification properties. Overall, this process could be readily adopted by cheese manufacturers to potentially create their own value-added, polysaccharide–whey protein-based products with the developed flocculates. Future research is warranted to fully characterize the polysaccharide composition and sensory attributes to better understand their potential applications in various formulated food products.

## Figures and Tables

**Table 1:** Yield, protein recovery, and selected mineral contents (dry weight basis) of polysaccharide-flocculated whey proteins. Each value represents the mean ± standard deviation. Data were analyzed by ANOVA (*p* < 0.05) and different lowercase letters within rows denote significant differences among treatments.

Parameters	Alginate	SKPE	Carrageenan
Yield of Flocculate (g/100 g)	1.66 ± 0.15 ^a^	0.98 ± 0.13 ^b^	1.22 ± 0.05 ^b^
Protein Concentration of Flocculate (%)	27.4 ± 3.3 ^b^	45.4 ± 4.9 ^a^	37.5 ± 1.3 ^a^
Whey Protein Recovery Efficiency (%)	57.5 ± 1.6 ^a^	56.2 ± 1.7 ^a^	57.9 ± 0.0 ^a^
Ca (mg/100 g)	957.1 ± 91.6 ^a^	812.5 ± 2.8 ^a^	411.9 ± 1.9 ^b^
K (mg/100 g)	1762.7 ± 187.4 ^a^	1158.3 ± 153.9 ^b^	2016.3 ± 9.1 ^a^
Mg (mg/100 g)	114.9 ± 11.5 ^a^	106.9 ± 8.2 ^a^	88.2 ± 0.5 ^a^
P (mg/100 g)	447.5 ± 45.8 ^a^	366.6 ± 23.7 ^a^	369.7 ± 2.9 ^a^
Na (mg/100 g)	834.2 ± 121.1 ^a^	333.0 ± 23.7 ^b^	428.1 ± 8.6 ^b^
Fe (mg/100 g)	1.9 ± 0.2 ^b^	5.9 ± 1.3 ^a^	2.2 ± 0.1 ^b^

**Table 2:** Color of the flocculate powders. BI represents browning index. Each value represents mean ± standard deviation. Data were analyzed by ANOVA (*p* < 0.05). Differences in lowercase letters within columns denote significant differences among treatments.

Sample	L*	a*	b*	BI	Flocculate Powder
Alginate	85.16 ± 0.08 ^a^	−0.52 ± 0.03 ^b^	13.78 ± 0.07 ^b^	−0.37 ± 0.02 ^b^	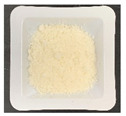
SKPE	50.83 ± 0.01 ^c^	5.66 ± 0.01 ^a^	32.69 ± 0.02 ^a^	8.11 ± 0.01 ^a^	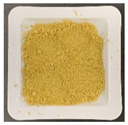
Carrageenan	82.56 ± 0.01 ^b^	−0.59 ± 0.00 ^c^	12.78 ± 0.02 ^c^	−0.45 ± 0.00 ^c^	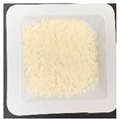

**Table 3:** Functionality and antioxidant activity of the flocculate powders. Each value represents the mean +/− standard deviation. Data were analyzed by ANOVA (*p* < 0.05) within each parameter. Differences in lowercase letters within parameters denote significant differences.

Parameters	Alginate	SKPE	Carrageenan
Water-Holding Capacity (g/g)	6.52 ± 0.47 ^a^	3.84 ± 0.23 ^b^	4.61 ± 0.18 ^b^
Oil-Holding Capacity (g/g)	6.08 ± 0.19 ^a^	4.00 ± 0.05 ^b^	6.17 ± 0.01 ^a^
Least Gelling Concentration % (*w*/*v*)	8.00	9.00	7.00
Emulsifying Activity (Abs)	0.54 ± 0.03 ^b^	0.74 ± 0.04 ^a^	0.79 ± 0.05 ^a^
Foaming Activity (% Foam Height)	33.75 ± 5.30 ^a^	14.60 ± 1.97 ^b^	17.32 ± 1.69 ^b^
Total Phenolic Content (mg GAE/g, dwb)	0.14 ± 0.00 ^b^	0.32 ± 0.02 ^a^	0.19 ± 0.06 ^b^
DPPH EC_50_ (mg/mL)	96.70 ± 6.10 ^a^	40.80 ± 1.60 ^c^	69.60 ± 2.60 ^b^

**Table 4:** Protein digestibility of the flocculate powders and freeze-dried whey. Each value represents the mean +/− standard deviation. Data were analyzed by ANOVA (*p* < 0.05) where lowercase letters denote significant differences.

Parameters	Whey	Alginate	SKPE	Carrageenan
Protein Digestibility (%)	98.64 ± 0.18 ^a^	97.88 ± 0.28 ^b^	94.29 ± 0.09 ^d^	95.57 ± 0.22 ^c^

## Data Availability

The original contributions presented in the study are included in the article; further inquiries can be directed to the corresponding author.

## References

[1] Tunick M., Thomas-Gahring A., Van Hekken D., Iandola S., Singh M., Qi P., Ukuku D., Mukhopadhyay S., Onwulata C., Tomasula P. (2016). Physical and chemical changes in whey protein concentrate stored at elevated temperature and humidity. J. Dairy Sci..

[2] Aguero R., Bringas E., San-Román M., Ortiz I., Ibáñez R. (2017). Membrane processes for whey protein separation and purification. A review. Curr. Org. Chem..

[3] Whitney M. (2023). Personal communication.

[4] Pattberg P., Bäckstrand K. (2023). Enhancing the achievement of the SDGs: Lessons learned at the half-way point of the 2030 Agenda. Int. Environ. Agreem..

[5] Capper J.L., Cady R.A. (2019). The effects of improved performance in the U.S. dairy cattle industry on environmental impacts between 2007 and 2017. J. Anim. Sci..

[6] Hoffman J.R., Falvo M.J. (2004). Protein—Which is best?. J. Sports Sci. Med..

[7] Panesar P.S., Kennedy J., Gandhi D., Bunko K. (2007). Bioutilisation of whey for lactic acid production. Food Chem..

[8] Lievore P., Simões D., Silva K., Drunkler N., Barana A., Nogueira A., Demiate I. (2015). Chemical characterisation and application of acid whey in fermented milk. J. Food Sci. Technol..

[9] Fox P.F., Guinee T., Cogan T., McSweeney W. (2016). Whey and whey products. Fundamentals of Cheese Science.

[10] Dragone G., Mussatto S., Oliveira J., Teixeira K. (2009). Characterisation of volatile compounds in an alcoholic beverage produced by whey fermentation. Food Chem..

[11] de Moura S., Lollo P., Morato P., Carneiro E., Amaya-Farfan J. (2013). Whey protein hydrolysate enhances the exercise-induced heat shock protein (HSP70) response in rats. Food Chem..

[12] Boirie Y., Dangin M., Gachon P., Vasson M., Maubois J., Beaufrère B. (1997). Slow and fast dietary proteins differently modulate postprandial protein accretion. Proc. Natl. Acad. Sci. USA.

[13] Juang R., Lin K. (2004). Flux recovery in the ultrafiltration of suspended solutions with ultrasound. J. Membr. Sci..

[14] Anandharamakrishnan C., Rielly C.D., Stapley A. (2007). Effects of process variables on the denaturation of whey proteins during spray drying. Drying Technol..

[15] Baker C., McKenzie K. (2005). Energy consumption of industrial spray dryers. Drying Technol..

[16] Zandona E., Blažić M., Jambrak A. (2021). Whey utilization: Sustainable uses and environmental approach. Food Technol. Biotechnol..

[17] Pal P., Nayak J. (2016). Development and analysis of a sustainable technology in manufacturing acetic acid and whey protein from waste cheese whey. J. Clean. Prod..

[18] Liu F., Jiang Y., Du B., Chai Z., Jiao T., Zhang C., Ren F., Leng X. (2013). Design and Characterization of Controlled-Release Edible Packaging Films Prepared with Synergistic Whey-Protein Polysaccharide Complexes. J. Agric. Food Chem..

[19] Yoo S., Krochta J.M. (2011). Whey Protein–Polysaccharide Blended Edible Film Formation and Barrier, Tensile, Thermal, and Transparency Properties. J. Sci. Food Agric..

[20] Loyeau P.A., Spotti M.J., Vinderola G., Carrara C.R. (2021). Encapsulation of Potential Probiotic and Canola Oil Through Emulsification and Ionotropic Gelation, Using Protein/Polysaccharides Maillard Conjugates as Emulsifiers. LWT.

[21] Combrinck J., Otto A., du Plessis J. (2014). Whey Protein/Polysaccharide-Stabilized Emulsions: Effect of Polymer Type and pH on Release and Topical Delivery of Salicylic Acid. AAPS PharmSciTech.

[22] de Jong S., Klok H.J., van de Velde F. (2009). The Mechanism Behind Microstructure Formation in Mixed Whey Protein–Polysaccharide Cold-Set Gels. Food Hydrocoll..

[23] Forghani B., Bordes R., Ström A., Undeland I. (2020). Recovery of a protein-rich biomass from shrimp (*Pandalus borealis*) boiling water: A colloidal study. Food Chem..

[24] Hao Y., Liu L., Feng G., Jin Q., Zou X., Xie D., Wang X. (2016). Polysaccharides as coagulants for the recovery of protein in fish meal wastewater. J. Aquat. Food Prod. Technol..

[25] Moreira A., Gaspar D., Ferreira S., Correia A., Vilanova M., Perrineau M., Kerrison P. (2023). Water-soluble *Saccharina latissima* polysaccharides and relation of their structural characteristics with in vitro immunostimulatory and hypocholesterolemic activities. Mar. Drugs.

[26] Ye A. (2008). Complexation between milk proteins and polysaccharides via electrostatic interaction: Principles and applications—A review. Int. J. Food Sci. Technol..

[27] Phomkaivon N., Pongponpai P., Kosawatpat P., Thongdang B., Panutai W. (2024). Extraction, characterisation and evaluation of antioxidant and probiotic growth potential of water-soluble polysaccharides from Ulva rigida macroalgae. Foods.

[28] Bradford M. (1976). A rapid and sensitive method for the quantitation of microgram quantities of protein utilizing the principle of protein-dye binding. Anal. Biochem..

[29] Evers J., Hughes C. (2002). Chemical analysis. Encyclopedia of Dairy Sciences.

[30] Lowry O.H., Rosebrough N.J., Farr A.L., Randall R.J. (1951). Protein measurement with the Folin phenol reagent. J. Biol. Chem..

[31] Shearer K.D. (1984). Changes in elemental composition of hatchery-reared rainbow trout, Salmo gairdneri, associated with growth and reproduction. Can. J. Fish. Aquat. Sci..

[32] Kasim R., Kasim M.U. (2015). Biochemical changes and color properties of fresh-cut green bean (*Phaseolus vulgaris* L. cv. gina) treated with calcium chloride during storage. Ciência Tecnol. Aliment..

[33] Tan E., Ying-Yuan N., Gan C. (2014). A comparative study of physicochemical characteristics and functionalities of pinto bean protein isolate (PBPI) against the soybean protein isolate (SPI) after the extraction optimization. Food Chem..

[34] Aydemir L., Yemenicioğlu A. (2013). Potential of Turkish kabuli type chickpea and green and red lentil cultivars as source of soy and animal origin functional protein alternatives. LWT—Food Sci. Technol..

[35] Islam M.R., Haque A.R., Kabir M.R., Hasan M.M., Khushe K.J., Hasan S.M.K. (2021). Fruit by-products: The potential natural sources of antioxidants and α-glucosidase inhibitors. J. Food Sci. Technol..

[36] Tang C., Yang X., Chen Z., Wu H., Peng Z. (2005). Physicochemical and structural characteristics of sodium caseinate biopolymers induced by microbial transglutaminase. J. Food Biochem..

[37] Blois M.S. (1958). Antioxidant determinations by the use of a stable free radical. Nature.

[38] Świeca M., Gawlik-Dziki U., Dziki D., Baraniak B., Czyż J. (2013). The influence of protein–flavonoid interactions on protein digestibility in vitro and the antioxidant quality of breads enriched with onion skin. Food Chem..

[39] Kim W., Wang Y., Vongsvivut J., Ye Q., Selomulya C. (2023). On surface composition and stability of β-carotene microcapsules comprising pea/whey protein complexes by synchrotron-FTIR microspectroscopy. Food Chem..

[40] Pelegrine D., Gomes T. (2012). Analysis of whey proteins solubility at high temperatures. Int. J. Food Eng..

[41] Modler H., Emmons D. (1977). Properties of whey protein concentrate prepared by heating under acidic conditions. J. Dairy Sci..

[42] De Wit J., van Kessel T. (1996). Effects of ionic strength on the solubility of whey protein products: A colloid chemical approach. Food Hydrocoll..

[43] Bajdik J., Makai Z., Berkesi O., Süvegh K., Marek T., Erős I., Pintye-Hódi K. (2009). Study of the effect of lactose on the structure of sodium alginate films. Carbohydr. Polym..

[44] McClements D. (2014). Nanoparticle- and Microparticle-Based Delivery Systems: Encapsulation, Protection and Release of Active Compounds.

[45] Liu D., Zhou P., Nicolai T. (2020). Effect of kappa carrageenan on acid-induced gelation of whey protein aggregates. Part I: Potentiometric titration, rheology and turbidity. Food Hydrocoll..

[46] Dickinson E. (1998). Stability and rheological implications of electrostatic milk protein–polysaccharide interactions. Trends Food Sci. Technol..

[47] Zhi J., Kilara A. (1998). Gelation of pH-aggregated whey protein isolate solution induced by heat, protease, calcium salt, and acidulant. J. Agric. Food Chem..

[48] Wright C., Wooton K., Twiss K., Newman E., Rasbury E.T. (2021). Boron isotope analysis reveals borate selectivity in seaweeds. Environ. Sci. Technol..

[49] Wegeberg S., Søndergaard J., Geertz-Hansen O. (2023). Elements and sugars in kelp and fucoid species in Greenland: Correlation and seasonality. Algal Res..

[50] Wong N.P., LaCroix D.E., McDonough F.E. (1978). Minerals in Whey and Whey Fractions. J. Dairy Sci..

[51] Premarathna A.D., Tuvikene R., Fernando P.H.P., Adhikari R., Perera M.C.N., Ranahewa T.H., Howlader M.M., Wangchuk P., Jayasooriya A.P., Rajapakse R.P.V.J. (2022). Comparative Analysis of Proximate Compositions, Mineral, and Functional Chemical Groups of 15 Different Seaweed Species. Sci. Rep..

[52] Zhao D., Yu D., Kim M., Gu M., Kim S., Pan C., Kim G., Chung D. (2019). Effects of temperature, light, and pH on the stability of fucoxanthin in an oil-in-water emulsion. Food Chem..

[53] Akomea-Frempong S., Skonberg D., Camire M.E., Perry J. (2021). Impact of blanching, freezing, and fermentation on physicochemical, microbial, and sensory quality of sugar kelp (*Saccharina latissima*). Foods.

[54] Baba W., Din S., Punoo H., Wani T., Ahmad M., Masoodi F. (2016). Comparison of cheese and paneer whey for production of a functional pineapple beverage: Nutraceutical properties and shelf life. J. Food Sci. Technol..

[55] Pérez-Mateos M., Solas T., Montero P. (2002). Carrageenans and alginate effects on properties of combined pressure and temperature in fish mince gels. Food Hydrocoll..

[56] Reid S.D., Fennema R.O., Damodaran S., Parkin L.K., Fennema R.O. (2008). Water and ice. Fennema’s Food Chemistry.

[57] Jiang Z., Bai X. (2022). Effects of polysaccharide concentrations on the formation and physical properties of emulsion-templated oleogels. Molecules.

[58] Wittmüss M., Amft J., Heyn T., Schwarz K. (2024). Oil binding capacity and related physicochemical properties of commercial plant protein products. Food Biosci..

[59] Ettoumi L., Mohamed C. (2015). Some physicochemical and functional properties of pea, chickpea, and lentil whole flours. Int. Food Res. J..

[60] Liao C., Chang C., Nagarajan D., Chen C., Chan J. (2021). Algae-derived hydrocolloids in foods: Applications and health-related issues. Bioengineered.

[61] Zhong H., Gao X., Cheng C., Liu C., Wang Q., Han X. (2020). The structural characteristics of seaweed polysaccharides and their application in gel drug delivery systems. Mar. Drugs.

[62] Shinoda R., Uchimura T. (2018). Evaluating the creaming of an emulsion via mass spectrometry and UV–Vis spectrophotometry. ACS Omega.

[63] Seta L., Baldino N., Gabriele D., Lupi F., de Cindio B. (2013). The influence of carrageenan on interfacial properties and short-term stability of milk whey protein emulsions. Food Hydrocoll..

[64] Fontes-Candia C., Ström A., Lopez-Sanchez P., López-Rubio A., Martínez-Sanz M. (2020). Rheological and structural characterization of carrageenan emulsion gels. Algal Res..

[65] Chang Y., McClements D. (2016). Influence of emulsifier type on the in vitro digestion of fish oil-in-water emulsions in the presence of an anionic marine polysaccharide (fucoidan): Caseinate, whey protein, lecithin, or Tween 80. Food Hydrocoll..

[66] Stone A.K., Nickerson M.T. (2012). Formation and functionality of whey protein isolate–(kappa-, iota-, and lambda-type) carrageenan electrostatic complexes. Food Hydrocoll..

[67] Djemaa I., Andrieux S., Auguste S., Jacomine L., Tarnowska M., Drenckhan-Andreatta W. (2022). One-step generation of alginate-based hydrogel foams using CO2 for simultaneous foaming and gelation. Gels.

[68] Nooshkam M., Varidi M., Alkobaisi F. (2023). Licorice extract/whey protein isolate/sodium alginate ternary complex-based bioactive food foams as a novel strategy to substitute fat and sugar in ice cream. Food Hydrocoll..

[69] Nooshkam M., Varidi M., Alkobeisi F. (2022). Bioactive food foams stabilized by licorice extract/whey protein isolate/sodium alginate ternary complexes. Food Hydrocoll..

[70] Żmudziński D., Ptaszek P., Kruk J., Kaczmarczyk K., Rożnowski W., Berski W., Ptaszek A., Grzesik M. (2014). The role of hydrocolloids in mechanical properties of fresh foams based on egg white proteins. J. Food Eng..

[71] Sappati P., Nayak B., VanWalsum G., Mulrey O. (2019). Combined effects of seasonal variation and drying methods on the physicochemical properties and antioxidant activity of sugar kelp (*Saccharina latissima*). J. Appl. Phycol..

[72] Peña-Ramos E., Xiong Y. (2003). Whey and soy protein hydrolysates inhibit lipid oxidation in cooked pork patties. Meat Sci..

[73] David S., Magram Klaiman M., Shpigelman A., Lesmes U. (2020). Addition of Anionic Polysaccharide Stabilizers Modulates In Vitro Digestive Proteolysis of a Chocolate Milk Drink in Adults and Children. Foods.

[74] Chen M., Guo L., Nsor-Atindana J., Goff H.D., Zhang W., Mao J., Zhong F. (2020). The Effect of Viscous Soluble Dietary Fiber on Nutrient Digestion and Metabolic Responses I: In Vitro Digestion Process. Food Hydrocoll..

[75] Bhowmick G.D., Hayes M. (2022). In Vitro Protein Digestibility of Selected Seaweeds. Foods.

[76] Borreani J., Llorca E., Larrea V., Hernando I. (2016). Adding Neutral or Anionic Hydrocolloids to Dairy Proteins under In Vitro Gastric Digestion Conditions. Food Hydrocoll..

[77] Gómez-Ordóñez E., Rupérez P. (2011). FTIR-ATR Spectroscopy as a Tool for Polysaccharide Identification in Edible Brown and Red Seaweeds. Food Hydrocoll..

[78] Martins M.S., Nascimento M.H., Barbosa L.L., Campos L.C.G., Singh M.N., Martin F.L., Romão W., Filgueiras P.R., Barauna V.G. (2022). Detection and Quantification Using ATR-FTIR Spectroscopy of Whey Protein Concentrate Adulteration with Wheat Flour. LWT.

[79] van der Ven C., Muresan S., Gruppen H., de Bont D.B.A., Merck K.B., Voragen A.G.J. (2002). FTIR Spectra of Whey and Casein Hydrolysates in Relation to Their Functional Properties. J. Agric. Food Chem..

[80] Paşcalău V., Popescu V., Popescu G.L., Dudescu M.C., Borodi G., Dinescu A.M., Moldovan M. (2013). Obtaining and Characterizing Alginate/k-Carrageenan Hydrogel Cross-Linked with Adipic Dihydrazide. Adv. Mater. Sci. Eng..

